# A novel ionic liquid-based approach for DNA and RNA extraction simplifies sample preparation for bacterial diagnostics

**DOI:** 10.1007/s00216-024-05615-z

**Published:** 2024-11-08

**Authors:** Johanna Kreuter, Katharina Bica-Schröder, Ádám M. Pálvölgyi, Rudolf Krska, Regina Sommer, Andreas H. Farnleitner, Claudia Kolm, Georg H. Reischer

**Affiliations:** 1 Institute of Chemical, Environmental and Bioscience Engineering, Working Area Molecular Diagnostics 166/5/3, IFA Tulln, TU Wien, Tulln, Austria; 2 Institute of Applied Synthetic Chemistry, Research Group for Sustainable Organic Synthesis and Catalysis, TU Wien, Vienna, Austria; 3 Department of Agrobiotechnology (IFA-Tulln), University of Natural Resources and Life Sciences Vienna (BOKU), Tulln, Austria; 4 Institute for Global Food Security, School of Biological Sciences, Queen’s University Belfast, Belfast, Northern Ireland UK; 5 Institute for Hygiene and Applied Immunology, Unit Water Hygiene, Medical University Vienna, Vienna, Austria; 6 Division Water Quality and Health, Karl Landsteiner University of Health Sciences, Krems, Austria; 7 Institute of Chemical, Environmental and Bioscience Engineering, Research Group for Microbiology and Molecular Diagnostics 166/5/3, TU Wien, Vienna, Austria; 8 ICC Interuniversity Cooperation Centre Water & Health,

**Keywords:** RNA extraction, DNA extraction, Ionic liquids, Periopathogens, Rapid methods, Molecular diagnostics

## Abstract

DNA- and RNA-based diagnostics play a pivotal role in accurately detecting and characterizing health-relevant bacteria, offering insights into bacterial presence, viability and treatment efficacy. Herein, we present the development of a novel extraction protocol for both DNA and RNA, designed to enable simple and rapid molecular diagnostics. The extraction method is based on the hydrophilic ionic liquid (IL) 1-ethyl-3-methylimidazolium acetate and silica-coated magnetic beads. First, we developed an IL-based cell lysis protocol for bacteria that operates at room temperature. Subsequently, we established a magnetic bead purification procedure to efficiently and reproducibly extract DNA and RNA from the IL-lysates. The IL not only lyses the cells, but also facilitates the adsorption of nucleic acids (NAs) onto the surface of the magnetic beads, eliminating the need for a chaotropic binding buffer and allowing for purification of NAs without significant effort and materials required. Lastly, we combined the cell lysis step and the purification step and evaluated the novel IL-based extraction method on periopathogenic bacterial cultures, comparing it to commercial DNA and RNA extraction kits via (RT)-qPCR. In comparison to the reference methods, the IL-based extraction protocol yielded similar or superior results. Furthermore, costs are lower, required materials and equipment are minimal and the process is fast (30 min), simple and automatable. These characteristics favour the developed method for use in routine and high-throughput testing as well as in point-of-care, on-site and low-resource settings, thereby advancing the field of molecular diagnostics.

**Graphical Abstract:**

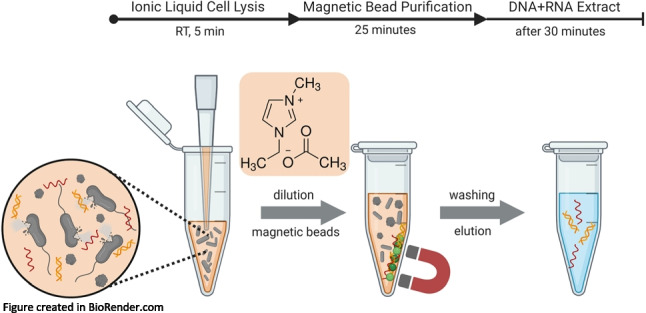

**Supplementary Information:**

The online version contains supplementary material available at 10.1007/s00216-024-05615-z.

## Introduction

In diagnostic microbiology, molecular methods can offer faster turnaround times as well as higher specificity and sensitivity in comparison to traditional culture-based approaches [1–5]. These advantages are also recognized in a guide on utilization of the microbiology laboratory for diagnosis of infectious diseases, advising on more nucleic acid amplification test-based diagnostics [6]. At present, commonly used molecular diagnostic techniques include (quantitative) PCR, isothermal amplification reaction, gene chip technology and high-throughput sequencing technology [7]. In most methods, DNA is the target analyte. For diagnostics of RNA viruses, assessing bacterial viability, monitoring antibiotic therapy, characterizing bacterial transcriptomic profiles, or increasing the detection sensitivity RNA-based diagnostics is necessary or preferred [8–13]. For both nucleic acids, a major obstacle preventing the progress of molecular diagnostics in routine and high-throughput analysis on the one hand, and in point-of-care, on-site, and low-resource settings on the other hand, is the requirement to extract these analytes from samples using time-consuming and/or complex protocols [14, 15]. Nucleic acid extraction procedures comprise cell lysis followed by the purification of nucleic acids from the lysate. The aim is to disrupt the cell envelope, denature proteins, remove chemicals and other biomolecules, and, finally, recover and concentrate the nucleic acids. Cell lysis is a critical part to ensure a suitable and successful extraction of nucleic acids and can be carried out enzymatically, chemically, thermally and/or mechanically [16]. Liquid–liquid extraction using hazardous chemicals such as phenol and chloroform, or commercial kits based on solid-phase extraction with silica columns are then used for purification, depending on the area of application and the sample matrix [16–18]. Although these protocols are well established and lead to high quality extracts, they are often very tedious, time-inefficient, and cost-intensive, or lack adequate and consistent yields, especially for readily degraded RNA [15]. Thus, more efficient and convenient nucleic acid extraction protocols are necessary to promote the implementation and further development of molecular diagnostics both inside and outside of the modern and well-equipped research laboratory.

Ionic liquids (ILs), which are organic salts that are liquid below temperatures of 100 °C, offer great potential for the development of simpler and more effective nucleic acid extraction methods. Hydrophobic ILs were used by Fister et al. and Fuchs-Telka et al. to isolate nucleic acids from viruses and Gram-negative bacteria [19, 20]. Emaus et al. applied magnetic hydrophobic ILs for isolating nucleic acids from whole blood and plants [21, 22]. On the other hand, soluble hydrophilic ILs were used by Garcia et al. and Ressmann et al. to efficiently lyse plants and meats [23, 24]. Martzy et al. successfully lysed Gram-negative and Gram-positive bacteria with the hydrophilic ILs 1-ethyl-3-methylimidazolium acetate ([C_2_mim][OAc]) and choline hexanoate ([Cho][Hex]) in 5 min at 65 °C [25]. However, they directly analysed released DNA from raw lysates without purification steps albeit after strong dilution. Without concentrating the nucleic acids via purification, direct analysis suffers from a higher limit of detection. Furthermore, inhibitors present in the sample matrix or the hydrophilic IL itself may have inhibiting effects on the downstream molecular diagnostics [26]. Silica-coated magnetic beads are excellent candidates for simple and fast nucleic acid purification. Similar to silica-membrane spin columns, nucleic acids in the sample lysate adsorb onto the surface of the beads in the presence of chaotropic salts, while other cell components in the lysate are removed via washing steps. A magnet applied to the side of the reaction tube collects the beads and allows for buffer exchange, thus eliminating the need for centrifugation [27, 28]. The nucleic acids are then eluted with a low ionic strength buffer [27–29]. Purification of nucleic acids via magnetic beads is easy to execute and virtually equipment-free, making it one of the best choices for automation, high-throughput applications, and high sample processivity [15, 27, 30].

The aim of this study was the development of a hydrophilic IL-based extraction method not only for DNA-based diagnostics but also for RNA-based diagnostics of bacteria that consistently generates high nucleic acid yields and can be carried out quickly and with minimal laboratory equipment. To this end, we selected periopathogenic bacteria as model organisms. In periodontology, molecular diagnostics rather than cultivation-based diagnostics are required due to difficulty in anaerobic culturing methods and long incubation times [31]. The development involved the evaluation of the best IL for rapid lysis of periopathogenic bacteria at room temperature and the combination with a straightforward magnetic bead purification protocol to efficiently and reproducibly extract both DNA and RNA from the IL-lysates. Since hydrophilic ILs have been reported to interact with biomass, nucleic acids, and other biomolecules, the compatibility with magnetic bead purification could not be readily assumed and had to be investigated [32–35]. Lastly, cell lysis and purification were combined to extract DNA and RNA from fresh periopathogenic bacterial cultures, comparing the performance to commercial DNA and RNA extraction kits quantitatively via (RT)-qPCR. Furthermore, we compared costs, process time and required materials and equipment.

## Materials and methods

### Hydrophilic ionic liquids used in this study

The two best performing ILs from Martzy et al., 1-ethyl-3-methylimidazolium acetate ([C_2_mim][OAc], MW = 170.21 g/mol) and choline hexanoate ([Cho][Hex], MW = 219.35 g/mol), were used in this study [25]. [C_2_mim][OAc] (purity > 95%, CAS 143314–17-4) was purchased from Iolitec (Heilbronn, Germany) as a viscous liquid. [Cho][Hex] was prepared according to literature procedures [36]: A freshly titrated solution of choline bicarbonate was charged into a 500-ml single-neck round-bottom flask and diluted with distilled water, followed by neutralization with hexanoic acid in a ratio 1:0.95 to avoid the presence of any excess acid. After the addition of acid was complete, the mixture was stirred at room temperature overnight. The mixture was concentrated *in vacuo*; then, it was further dried on high vacuum (0.4 mbar, room temperature) for 3 to 9 days. The product was obtained as an orange sludge. Reagents and solvents for the synthesis of [Cho][Hex] were used as received from Sigma-Aldrich (St. Louis, MO).

[C_2_mim][OAc] and [Cho][Hex] were used as a 90% w/w and 50% w/w solution in 10 mM Tris pH 8 buffer, respectively. The solutions were prepared by weighing the required amounts of IL and Tris buffer. With an approximate density of [C_2_mim][OAc] and Tris of 1.1 g/cm^3^ and 1.0 g/cm^3^ respectively, the 90% w/w solution corresponds to an 89% v/v solution. For [Cho][Hex], no volume percentages can be calculated due to its solid consistency. Unless otherwise specified, volume percentages are used in this paper.

### Bacterial strains used in this study

*Escherichia coli* type strain NCTC 9001 and five periopathogenic bacteria type strains were used in this study. *Aggregatibacter actinomycetemcomitans* ATCC 33384, *Porphyromonas gingivalis* ATCC 33277, *Treponema denticola* ATCC 35405, and *Prevotella intermedia* ATCC 25611 were obtained from the German Collection of Microorganisms and Cell Cultures (DSMZ). *Tannerella forsythia* ATCC 43037 was obtained from the Culture Collection University of Göteborg (CCUG).

*E. coli* was grown overnight in lysogeny broth (Merck, Darmstadt, Germany) at 37 °C. The overnight culture was diluted with fresh lysogeny broth to an optical density at 600 nm (OD_600_) of 0.1 and incubated to an OD_600_ of 1. *A. actinomycetemcomitans*, *P. gingivalis* and *P. intermedia* were grown anaerobically in tryptic soy broth (Merck) at 37 °C for 4 to 7 days. *T. forsythia* was grown anaerobically in ATCC NAM Medium at 37 °C for 10 days [37]. Anaerobic conditions were generated with the Anaerocult® system from Merck. *T. denticola* was purchased as an actively growing culture from DSMZ.

Cells from fresh culture were harvested by centrifugation (5 min, 4000 × g), washed, and resuspended in isotonic saline solution. 10 µl of the respective cell suspension was used for cell lysis and extraction experiments. Cell numbers were estimated via OD_600_. Total bacterial cell counts were obtained by epifluorescence microscopy. For this, bacterial cell suspensions were fixed with sterile-filtered paraformaldehyde (final concentration 0.8%) overnight at 4 °C. The fixed samples were filtered through 0.2-µm filters (Anodisc 25, Whatman, Germany) and stained with SYBR Gold. For SYBR Gold staining, the filters were placed in a petri dish on a drop (30 µl) of SYBR Gold (10,000 × concentrate in DMSO, diluted 400-fold in sterile deionized water) and kept in the dark for 15 min. After incubation, the filters were rinsed three times with a few drops of sterile-filtered Milli-Q water to remove excess dye, dried in the dark at room temperature and then mounted onto a microscopic slide with one drop of anti-fading mounting solution (Citifluor). Another drop of mounting solution was directly placed on the filter before adding the cover slip. Slides were examined with immersion oil using a Nikon Eclipse Ni microscope at 400 × or 1000 × magnification (Ex ~ 470, Em ~ 515) equipped with a Nikon DS-Qi2 camera.

### Ionic liquid-based extraction of DNA and RNA

#### Cell lysis

10 µl of a cell suspension was mixed with 90 µl of 90% w/w [C_2_mim][OAc] in 10 mM Tris pH 8 buffer and incubated at room temperature for 5 min. For direct analysis of released nucleic acids, the lysate was diluted with 10 mM Tris pH 8 buffer to overcome inhibitory effects caused by the ILs or cell components. For quantitative PCR (qPCR), the IL-lysate was diluted 1:20, and for reverse transcription qPCR (RT-qPCR), the lysate was diluted 1:10.

#### Magnetic bead purification

For purification of DNA and RNA, the undiluted [C_2_mim][OAc] lysate was mixed with 150 µl of SeraSil-Mag™ 400 silica-coated superparamagnetic beads (Cytiva, MA, USA). After adding 765 µl of 10 mM Tris pH 8 buffer, the mixture was vortexed and incubated on a thermomixer (24 °C, 10 min, 1400 rpm) to aid binding of nucleic acids to the beads. The reaction tube was placed into a magnetic separation rack and allowed to sit for 30 to 60 s for the collection of beads. The supernatant was discarded. After a wash step with 500 µl of 70% ethanol in 10 mM Tris pH 8 buffer, the beads were air-dried for 10 to 15 min. Elution was performed with 100 µl of TE buffer (10 mM Tris–HCl, 1 mM EDTA, pH 8.0), incubating in a thermomixer (65 °C, 3 min, 1400 rpm).

#### Reference extractions of DNA and RNA

The QIAamp DNA Mini Kit from QIAGEN (Hilden, Germany) and the Monarch Total RNA Miniprep Kit from NEB (Frankfurt, Germany) were used as reference methods for the extraction of DNA and RNA, respectively. The extraction procedures were carried out according to the manufacturers’ instructions for Gram-negative bacteria. In the QIAGEN Kit, cell lysis was achieved by adding proteinase K and detergent and incubating 1 h at 56 °C. In the NEB Kit, lysis was performed with lysozyme, incubating for 5 min at room temperature, and adding a chaotropic salt. Both kits used spin columns for purification. For RNA extraction on-column DNase digestion was performed.

### Quantification of bacterial DNA and RNA using quantitative PCR and reverse transcription quantitative PCR

Bacterial DNA in the lysis, purification and extraction experiments was quantified with a qPCR assay targeting the V1-V2 region of the 16S rRNA gene with primer binding sites universal to all bacteria (denoted as 16S-qPCR) [38]. The qPCR reactions were carried out in a total reaction volume of 15 µl containing each primer at a concentration of 200 nM (Merck) (see Supplementary Table S1 for oligonucleotide sequences), 7.5 µl KAPA™ SYBR® Fast qPCR Master Mix 2x (Peqlab, Erlangen, Germany) and 2.5 µl sample. The assay was performed on a qTOWER^3^ G real-time thermocycler (Analytik Jena, Jena, Germany) according to the following temperature protocol: 3 min at 95 °C, followed by 40 cycles of 30 s at 95 °C, 30 s at 57 °C and 1 min at 72 °C, respectively, and 2 min at 72 °C. Unless otherwise stated, qPCR reactions were carried out in duplicates. To rule out qPCR inhibition, samples were measured in multiple dilutions. The calibration curve was created by using a dilution series of DNA plasmid solution containing a known number of copies of the target 16S rRNA gene fragment. DNA templates used for qPCR calibration curves were quantified via PicoGreen measurements (Thermo Fisher Scientific, Vienna, Austria). No-template-controls (NTCs) were included in each qPCR run. Due to *E. coli* DNA residues in the polymerase, a small number of target copies are detected in each NTC. Since these numbers are several orders of magnitudes lower than those in the actual samples, runs were accepted if the NTCs contained less than 100 copies of the 16S rRNA target per reaction.

*P. intermedia* DNA was additionally quantified with a qPCR assay targeting a region of the *P. intermedia* 16S rRNA gene (denoted as PI qPCR) [39]. The qPCR reactions were carried out in a total reaction volume of 15 µl containing each primer at a concentration of 500 nM (Merck), a probe at a concentration of 200 nM (Merck) (see Supplementary Table S1 for oligonucleotide sequences), 7.5 µl KAPA™ Probe qPCR Master Mix 2x (Peqlab) and 2.5 µl sample. The assay was also performed on a qTOWER^3^ G thermocycler according to the following temperature protocol: 3 min at 95 °C, followed by 45 cycles of 15 s at 95 °C and 45 s at 56 °C. Unless otherwise stated, qPCR reactions were carried out in duplicates. To rule out qPCR inhibition, samples were measured in multiple dilutions. The calibration curve was created by using a dilution series of an artificial double-stranded DNA standard containing a known number of copies. DNA templates used for qPCR calibration curves were quantified via PicoGreen measurements (Thermo Fisher Scientific). NTCs were included in each qPCR run. Data were only accepted when all NTCs of a run were negative.

RNA was analysed via a two-step RT-qPCR workflow using the LunaScript RT SuperMix (NEB) with random priming for first strand complementary DNA (cDNA) synthesis according to the manufacturers’ protocol. 1 µl of RNA sample was used for reverse transcription. RT-qPCR was validated by measuring an RNA dilution series; the results were linear over six orders of magnitude. No-RT controls and NTCs were included in each experiment. Subsequent quantification of cDNA was performed via qPCR assays as explained above.

### Used software

Calculations were performed in Excel. Tables and diagrams were generated in Excel. Additional figures were generated in BioRender.

## Results and discussion

### Optimization of the IL-based cell lysis protocol

**Fig. 1:**
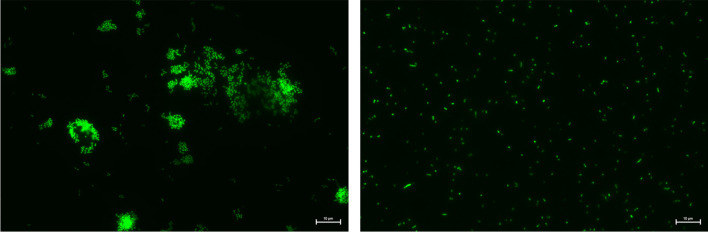
*P. intermedia* cells incubated with Tris buffer (left) and 90% [C_2_mim][OAc] (right) for 5 min at room temperature, filtered on a polycarbonate 0.2 µm filter, stained with SYBR gold, and imaged under an epifluorescence microscope (scale bar: 10 µm). A dissolution of cell aggregates can be observed in the IL-treated sample

In a previous study, Martzy et al. achieved bacterial cell lysis using 90% w/w (89% v/v) [C_2_mim][OAc] or 50% w/w [Cho][Hex] at 65 °C within 5 min [25]. We intended to simplify the protocol by eliminating the need for heating. To this end, we evaluated the lysis performance of these ILs at room temperature with *Escherichia coli* type strain NCTC 9001 and *Prevotella intermedia* type strain ATCC 25611. *P. intermedia* was selected as a target due to its tendency to form robust biofilms and presumed resistance to cell lysis. The test procedure involved preparing 10 µl of a cell suspension in isotonic saline solution, adding 90 µl of 90% w/w [C_2_mim][OAc] or 50% w/w [Cho][Hex], and incubating at room temperature for 5 min. Lysis performance was determined by measuring the released nucleic acids via qPCR. To mitigate inhibitory effects of the ILs, lysates were diluted 1:20 with Tris buffer [25]. To remove non-lysed cells, diluted lysates were filtered through a 0.22-µm PVDF syringe filter. Filtering ensured that only released nucleic acids were quantified. Intact cells carried over to qPCR might be disrupted by thermal cell lysis during the 95 °C denaturation step in the temperature protocol and bias the result of the lysis experiment. This approach allowed to assess the amount of nucleic acids released by incubation with the IL, which is available for subsequent direct analysis with methods avoiding high temperatures such as hybridisation, isothermal amplification, or reverse transcription as well as for further purification steps. Control experiments were performed using 10 mM Tris pH 8 buffer instead of ILs, with similar post-lysis handling to ensure consistency in measuring nucleic acid release. As a reference for lysis performance, kit-based DNA extraction (QIAamp DNA Mini kit) was performed. We calculated relative nucleic acid quantities between reference (extraction kit) and lysates and termed them lysis rates. For *E. coli* cells, the lysis rates of [C_2_mim][OAc] and [Cho][Hex] were 76% and 25%, respectively, compared to the yield of the kit (Table 1). Lysis in Tris buffer controls amounted to only 1.5%. For *P. intermedia* cells, the lysis rate of [C_2_mim][OAc] was also superior (160%) to that of [Cho][Hex] (12%) (Table 1), while Tris buffer negative controls had a 6.4% lysis rate. Furthermore, epifluorescence microscopy was used to visualize *P. intermedia* cell aggregates upon incubation with [C_2_mim][OAc] and revealed the disaggregation of cells, although morphological changes in the cell envelope were not discernible at the microscopy’s magnification level (Fig. 1).

**Table 1:** Lysis rates for tested bacterial strains, compared to DNA extraction with kit as 100%. Data shown are mean values from three biological replicates

Sample	Mean lysis rate [%]	Min [%]	Max [%]
*E. coli* [C_2_mim][OAc]	**76**	68	92
*E. coli* [Cho][Hex]	**25**	21	32
*E. coli* Tris buffer control	**1.5**	1.2	2.1
*P. intermedia* [C_2_mim][OAc]	**160**	114	241
*P. intermedia* [Cho][Hex]	**12**	8	18
*P. intermedia* Tris buffer control	**6.4**	5.0	8.8
*A. actinomycetemcomitans* [C_2_mim][OAc]	**127**	108	149
*A. actinomycetemcomitans* Tris buffer control	**3.0**	2.3	3.8
*P. gingivalis* [C_2_mim][OAc]	**119**	89	161
*P. gingivalis* Tris buffer control	**10**	8.7	13
*T. denticola* [C_2_mim][OAc]	**90**	72	102
*T. denticola* Tris buffer control	**1.6**	1.0	2.2
*T. forsythia* [C_2_mim][OAc]	**44**	36	53
*T. forsythia* Tris buffer control	**0.3**	0.2	0.5

We then assessed the lysis rate of [C_2_mim][OAc] at room temperature for the other periopathogens (Table 1). Lysis rates around 100% were achieved for almost all strains, with *T. forsythia* showing greater resistance to lysis. Again, negative controls with Tris buffer indicated no significant cell lysis.

We expected lysis performance for RNA analysis to be comparable to DNA experiments because the entire intercellular content is released during cell lysis. To confirm this, we performed RT-qPCR of 1:10 dilutions of [C_2_mim][OAc] lysates. Rates between 136 and 773% (Supplementary Table S2) compared to RNA extraction with a kit (Monarch Total RNA Miniprep Kit) confirmed the highly efficient release of RNA for all tested bacterial strains and suggested that RNA is stable in the IL. Interestingly, the lysis rates of the Tris buffer negative controls also increased. This difference between DNA and RNA lysis rates might be attributed to variations in kit performance. No DNase digestion was performed in the IL-lysates. A comparison of the 16S rDNA and 16S rRNA content in the IL-lysates revealed that the amount of rRNA was 50–500 times higher than the amount of rDNA, depending on cell activity. Therefore, the error introduced into rRNA (cDNA) results due to rDNA present in the lysate was negligible.

### Establishment of a magnetic bead purification protocol for IL-lysates

In order to transform the simple cell lysis method into a full DNA and RNA extraction procedure, we intended to establish a magnetic bead-based purification protocol to efficiently extract nucleic acids from our IL-lysates. The lysates consisted of a 100 µl mixture containing lysed cells and 80% [C_2_mim][OAc]. We selected SeraSil-Mag silica-coated superparamagnetic particles with a sub-microscale diameter of 400 nm (Cytiva) for our experiments, because they are compatible with chaotropic salt chemistry and relatively inexpensive when compared to products from other suppliers.

For method establishment, we first evaluated the purification performance by spiking a known amount of *E. coli* genomic DNA (gDNA) into an 80% [C_2_mim][OAc] solution to a total volume of 100 µl and measuring the DNA recovered by magnetic bead purification via qPCR. Thereby, the purification performance in the presence of the IL could be assessed independently of cell lysis. As positive controls, the same *E. coli* gDNA was spiked into 10 mM Tris pH 8 buffer and processed identically to the IL-DNA samples. Tris buffer did not interfere with the chaotropic binding mechanism of the beads. Relative quantities were calculated between the *E. coli* gDNA spike and the gDNA recovered after purification. These were termed purification rates.

The initial protocol used 15 µl of magnetic beads and 4 M guanidinium chloride (Gua-HCl) as a chaotropic salt for binding. Due to possible interactions between IL, chaotropic salt, nucleic acids, and beads, the purification protocol was tested with different dilutions of the 80% IL-DNA sample (undiluted, 1:2, 1:3.6 and 1:5). After the corresponding dilution with Tris buffer, 6 M Gua-HCl binding buffer was added to achieve a final concentration of 4 M Gua-HCl, followed by the addition of 15 µl magnetic beads, resulting in final IL concentrations of 25%, 13%, 7%, and 5%, respectively. The reaction was incubated for 10 min at room temperature and shaken at 1400 rpm. The beads were washed three times with 70% ethanol in 10 mM Tris pH 8 buffer and dried, and finally, DNA was eluted with 100 µl TE buffer. The results indicated that no substantial amount of DNA could be recovered at any concentration of [C_2_mim][OAc] in contact with the magnetic beads (Table 2). Repeated elution as well as dilution of the extracts to avoid possible inhibition of the qPCR by IL residues in the extract did not lead to an overall increase in the purification rates. The best performance was observed at 7% and 5% IL, with a 14% mean purification rate. The rates for the positive controls containing Tris buffer instead of [C_2_mim][OAc] were between 115 and 56%, decreasing with increasing sample volume. Higher dilutions of the IL-DNA mixture were not tested, as the total sample volume was limited by the 1.5-ml reaction tube and the recovery for the positive controls decreased with higher sample volumes.

**Table 2:** Purification rates of spiked *E. coli* gDNA for IL-DNA samples with different dilutions resulting in different concentrations of the IL in contact with the magnetic beads. The Tris-DNA samples (0%) were similarly diluted and served as positive controls. The concentration of the chaotropic salt Gua-HCl was 4 M. Purification rates shown are mean values from three biological replicates

Final concentration [C_2_mim][OAc]	Total volume [µl]	Mean purification rate [%]	Min [%]	Max [%]
25%	315	**7.4**	5.6	12
0% (Tris buffer, positive control)	315	**109**	87	138
13%	615	**10**	7.8	12
0% (Tris buffer, positive control)	615	**115**	99	140
7%	1095	**14**	11	19
0% (Tris buffer, positive control)	1095	**83**	69	106
5%	1515	**14**	11	18
0% (Tris buffer, positive control)	1515	**56**	45	67

**Fig. 2:**
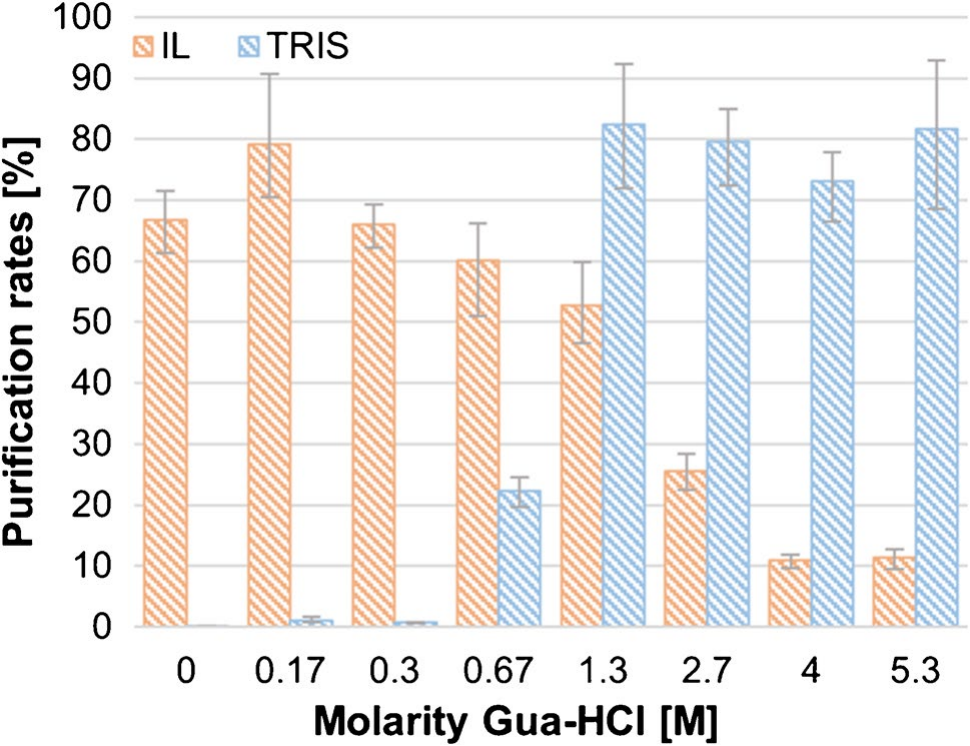
Mean purification rates of spiked *E. coli* gDNA in the presence of 7% [C_2_mim][OAc] (orange) and Tris buffer (blue), respectively, varying the molarity of Gua-HCl (0 to 5.3 M). 0 M corresponds to the use of water. Values shown are mean values from three biological replicates. The whiskers indicate the highest and lowest values

In following experiments, we diluted the samples to a final concentration of 7% IL and varied the molarity of Gua-HCl in the binding buffer. Surprisingly, the purification rates for the IL-DNA samples increased as the molarity of Gua-HCl decreased (Fig. 2, orange bars). The highest purification rates were achieved in the presence of 0.17 M Gua-HCl, indicating that the IL effectively facilitated adsorption of the DNA onto the bead surface without Gua-HCl. Presumably, in the presence of IL and high concentrations of Gua-HCl, an unfavourable reaction occured between imidazolium, guanidinium and DNA, preventing purification. In contrast, when Tris buffer was used instead of [C_2_mim][OAc], purification rates decreased with decreasing molarity of the chaotropic binding buffer, as expected (Fig. 2, blue bars). Consequently, we fully omitted the Gua-HCl-based binding buffer in favour of a straightforward protocol.

In order to determine the optimal concentration of [C_2_mim][OAc] for purification, different dilutions of the 80% IL-DNA samples were tested again. A concentration of 7% [C_2_mim][OAc] yielded the best purification rate of about 80% (Table 3), corresponding to a dilution of the IL-DNA sample with Tris buffer of approx. 1:10.8, followed by the addition of 15 µl beads. To simplify handling, the dilution was adjusted to 1:10 for further experiments.

**Table 3:** Purification rates of spiked *E. coli* gDNA for IL-DNA samples with different dilutions resulting in different concentrations of the IL in contact with the magnetic beads. The Gua-HCl binding buffer was fully omitted. Purification rates shown are mean values from three biological replicates

Final concentration [C_2_mim][OAc]	Mean purification rate [%]	Min [%]	Max [%]
32%	**18**	14	21
16%	**34**	21	72
7%	**78**	72	85
4%	**76**	60	91

**Fig. 3:**
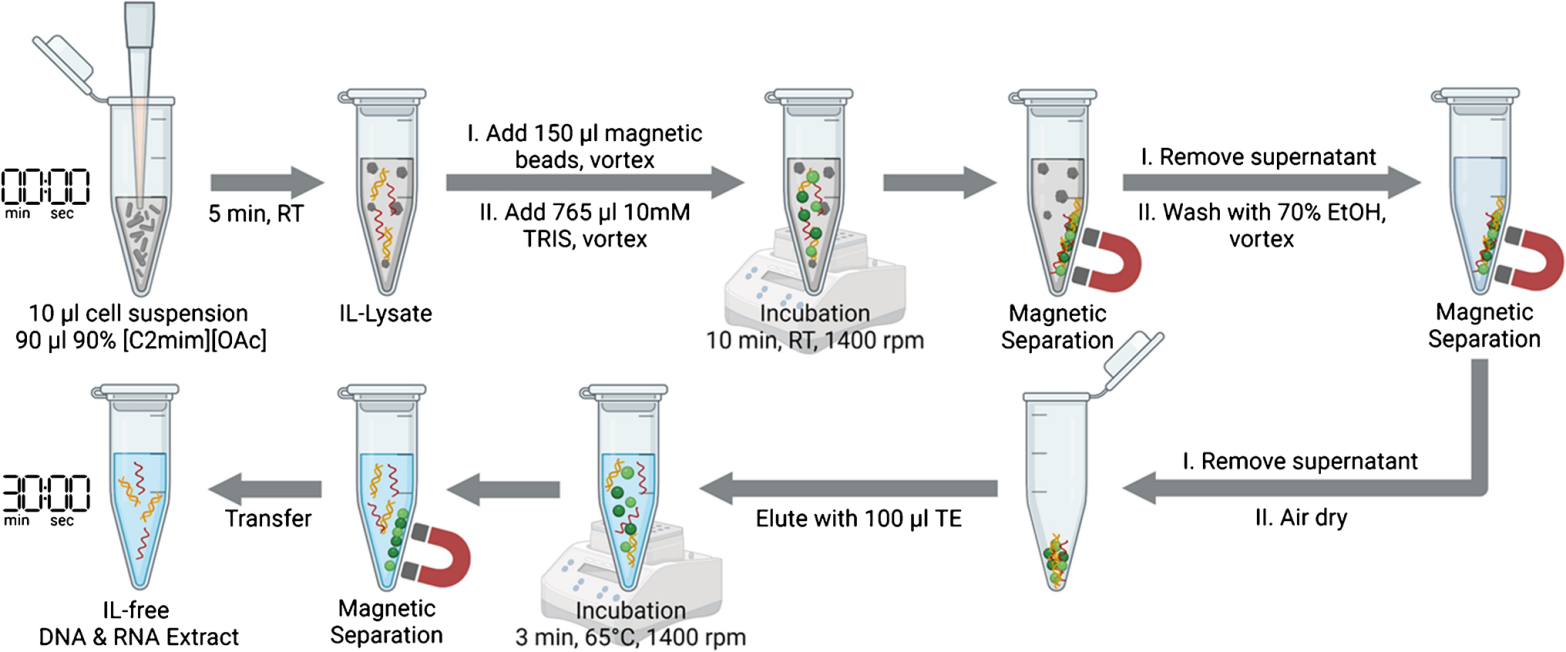
Final workflow of the developed IL-based extraction protocol for DNA and RNA. The processing time for the entire procedure is about 30 min, hands-on-time sum up to around 2 min. Figure created in BioRender.com

Lastly, possible interference of cell debris present in IL-lysates with the magnetic bead purification was investigated by testing the protocol on cell IL-lysates. For this, 10 µl of a *P. intermedia* or *E. coli* cell suspension was lysed with 90 µl 90% w/w [C_2_mim][OAc] for 5 min at room temperature, followed by 1:10 dilution with Tris buffer and the addition of 15 µl magnetic beads. However, the initial purification rates for DNA from *P. intermedia* and *E. coli* lysates were only around 30% compared to the highly diluted crude IL-lysate. The performance was improved by increasing the volume of magnetic beads to 150 µl and changing the order of magnetic bead addition. Additionally, the number of washing steps was optimized for a faster protocol. Figure 3 depicts the final proposed IL-based extraction protocol for bacterial cells. Using the improved purification workflow, we achieved a DNA purification rate of 93% for *E. coli* compared to the crude lysate. For *P. intermedia* cells, the purification rate was around 50%.

For evaluation of the purification performance for RNA, a known amount of *P. intermedia* total RNA was first spiked into an 80% [C_2_mim][OAc] solution to a total volume of 100 µl and purified according to the final workflow (Fig. 3), starting with the addition of 150 µl magnetic beads. The amount of recovered RNA was measured via RT-qPCR and compared to the RNA spike. Purification rates of around 100% could be achieved. Subsequently, experiments with *P. intermedia* cells were performed. The samples were processed according to the final workflow (Fig. 3). A purification rate of 100% RNA compared to the crude lysate was achieved and indicated good purification performance for RNA. The results show that the developed protocol is suitable for the purification of both DNA and RNA from IL-lysates.

Although the purification rates of the developed purification protocol were below 100% for DNA, the purification step had a concentrating effect and increased the detection limit. In theory, at 100% purification rate, the detection limit would increase by a factor of 20 compared to the 1:20 diluted crude lysate. At 50%, the detection limit would still increase by a factor of 10. As a result, without the purification step, the diluted lysate would quickly fall below the detection limit when the cell count in the sample decreases. For RNA, at 100% purification rate, the detection limit would increase by a factor of 10 compared to the 1:10 diluted crude lysate.

### DNA and RNA extraction from health-relevant *bacteria*

**Fig. 4:**
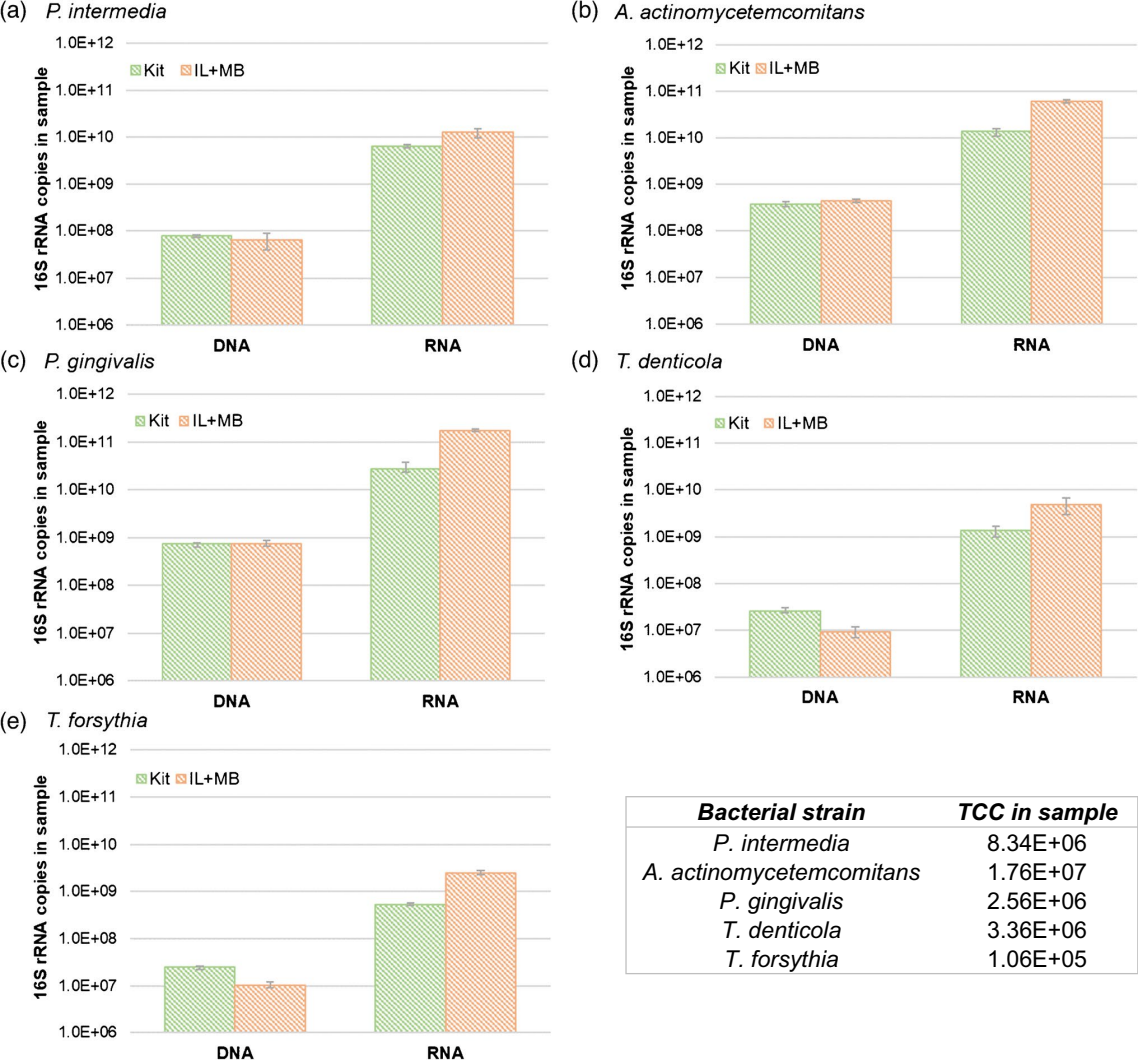
16S rRNA (gene) copies in the sample, extracted with three extraction methods (QIAGEN Kit for DNA, NEB Kit for RNA, IL-based magnetic bead extraction protocol (IL + MB) for DNA and RNA), of five periopathogenic bacterial strains. All strains were extracted three times with each individual method. Values shown are mean values. The whiskers indicate the highest and lowest values. The table in the lower right corner shows the number of cells (total cell count, TCC) used for each extraction run

Following the successful establishment of a cell lysis and purification protocol, both steps were combined to extract DNA and RNA from five freshly grown periopathogenic bacterial strains: *P. intermedia*, *A. actinomycetemcomitans*, *P. gingivalis*, *T. denticola* and *T. forsythia*. Anaerobic cultivation of the periopathogens was incredibly challenging due to the long processing time and complex media. Notably, *T. denticola* could not be cultivated and had to be purchased as an actively growing culture. However, cultivation was necessary to obtain samples with a high and defined cell number. All strains were imaged under epifluorescence microscopy (see Supplementary Fig. S1) and the number of cells used for the extractions was determined. We compared the yield of our new DNA/RNA extraction method to two popular commercial extraction kits (Fig. 4). For DNA, our extraction method yielded results equivalent to that of the commercial DNA extraction kit (QIAamp DNA Mini kit, QIAGEN) for *P. intermedia*, *A. actinomycetemcomitans* and *P. gingivalis*. For *T. denticola* and *T. forsythia*, only a 40% yield could be achieved compared to the commercial kit. For RNA, the performance of the new method was superior to the RNA extraction kit (Monarch Total RNA Miniprep Kit, NEB) for all tested strains. Since the new method isolates both DNA and RNA from cells, the corresponding extracts were not DNase digested. However, as mentioned before, rDNA and rRNA concentrations differed by a factor of 50 to 500. Therefore, we assumed that the rDNA had a negligible impact on the concentration of rRNA (or rather corresponding cDNA) measured.

### Comparison and benefits of novel method

This is the first report of bacterial DNA and RNA purified from hydrophilic IL-lysates. Fister et al. and Fuchs-Telka et al. have previously used hydrophobic ILs for the isolation of nucleic acids from viruses and Gram-negative bacteria by back-extracting the nucleic acids from the IL-lysate with water [19, 20]. Lysis of Gram-negative bacteria with hydrophobic ILs was performed at extreme process parameters (80–180 °C). Emaus et al. used magnetic hydrophobic ILs for the simultaneous cell lysis and DNA purification from whole blood and plants [21, 22].

Our developed extraction method could keep up with or exceed the performance of two widely used extraction kits for DNA and RNA. Our method is carried out in a single reaction tube and simultaneously extracts both DNA and RNA from bacterial cells. The major advantages are its simplicity, cost-effectiveness, and the limited amount of equipment, chemicals, and time required (Table 4). Since the workflow is straightforward and only requires minimal hands-on-time, it allows for high sample throughput and automation. Limited sample handling and change of reaction vessels make this a “one pot” method, decreasing the risk of yield loss or cross contamination during extraction. In contrast, the commercial RNA extraction kit includes many pipetting steps, which might explain the poor yield. Additionally, the developed method requires fewer consumables, like pipette tips and reaction tubes, thus reducing the environmental footprint. The chemicals used are non-hazardous and can easily be stored at room temperature [40]. Our method also avoids high temperatures or mechanical shear stress that might fragment nucleic acids. This fact will positively affect the properties of the extracted DNA and RNA in long-read sequencing applications. Economically, the cost per extraction with [C_2_mim][OAc] and magnetic beads (IL + MB) is approximately 2€ for DNA and RNA together, compared to 4.94€ for the commercial DNA kit (QIAGEN) and 5.58€ for the RNA kit (NEB), depending on the number of preps in the kit.

**Table 4:** Comparison of extraction methods

	QIAGEN Kit	NEB Kit	IL + MB
Target nucleic acid	DNA	RNA	DNA + RNA
Cost per extraction	4.94€	5.58€	≈2€
Processing time	≈80 min	≈30 min	≈30 min
Equipment	Centrifuge Thermoshaker Silica columns	Centrifuge Silica columns	Thermoshaker Magnetic beads + rack
Solutions and buffers	Proteinase K 96% ethanol Buffer 1 Buffer 2 Wash buffer 1 Wash buffer 2 Elution buffer	Lysozyme 96% ethanol Nuclease-free water Buffer 1 Buffer 2 Wash buffer	[C_2_mim][OAc] Tris buffer 70% ethanol TE buffer

In the broader context of diagnostic microbiology in clinical and environmental settings, the labour-intensive and time-consuming cultivation of periopathogens exemplifies the importance of efficient molecular methods to complement or replace culture-based methods. *Prevotella* spp. are not only relevant in periodontitis, but also associated with other human infections such as chronic osteomyelitis, bite-related infections, rheumatoid arthritis and intestinal diseases [41]. Despite the limited selection of bacteria tested, we expect our method to be applicable to many other Gram-negative health-relevant bacteria. Nonetheless, it is necessary to evaluate the performance of the method in relation to other bacterial targets and sample types. Moreover, further validation and integration into molecular workflows and clinical practice are warranted to realize its full potential.

## Conclusion

This study presents a novel nucleic acid extraction method for both bacterial DNA and RNA, based on the hydrophilic IL [C_2_mim][OAc] and magnetic beads. The developed method offers significant improvements in terms of simplicity, cost-effectiveness and amount of equipment, chemicals, and time required compared to commercial kit solutions. [C_2_mim][OAc] is commercially available and can be purchased inexpensively with consistent quality. The IL is capable of efficiently lysing tested bacterial strains at room temperature, providing DNA and RNA for subsequent diagnostics. We also observed the dissolution of cell aggregates upon incubation with [C_2_mim][OAc]. This property could potentially improve processing of clinical or environmental samples containing biofilms (e.g. subgingival plaque samples, sputum, sediments). Further application to other targets strongly depends on the composition of the cell envelope. Human and animal cells are rather fragile and might be easily lysed and extracted with our method. Moreover, [C_2_mim][OAc] enables the adsorption of RNA and DNA onto the silica surface of magnetic beads, simplifying the purification procedure and eliminating the need for an additional binding buffer. This property did not apply to [Cho][Hex]. However, other hydrophilic ILs may possess similar favourable properties for lysis and purification.

Our method provides both DNA and RNA for downstream molecular diagnostics. In contrast to DNA, RNA allows assessment of bacterial viability, monitoring of antibiotic therapy, characterisation of bacterial transcriptomic profiles or increased detection sensitivity. Depending on the specific needs, the crude IL-lysate or the purified extract can be used for molecular diagnostics. For instance, in low-resource, on-site and point-of-care settings, rapid lysis at room temperature followed by dilution in combination with suitable diagnostics (isothermal amplification, hybridisation) provides a quick and straightforward way to achieve a direct result [42]. Exceptionally low cost and limited equipment, chemicals, and time required make this approach highly suitable for such settings. However, due to the dilution step required to avoid inhibition of or interference with the subsequent detection method, a loss of detection limit has to be accepted. Moreover, storability of the crude lysate is limited. For applications requiring purified and concentrated DNA or RNA, a combination with magnetic bead purification is possible without significant effort. The extraction method is then suitable for high-throughput and routine laboratory analysis providing simplicity and the possibility for automation in various diagnostic and research settings.

## Supplementary Information

Below is the link to the electronic supplementary material.

Supplementary file1 (DOCX 1558 KB)

Supplementary file2 (XLSX 32 KB)
