# Supplementary material for: A novel ionic liquid-based approach for DNA and RNA extraction simplifies sample preparation for bacterial diagnostics
Source: Anal Bioanal Chem. 2024 Nov 8;416(29):7109–20. doi: 10.1007/s00216-024-05615-z (PMC11579088; doi:10.1007/s00216-024-05615-z)
Supplement: Supplementary file 1 — Supplementary file1 (DOCX 1558 KB) [file 216_2024_5615_MOESM1_ESM.docx]

**Analytical and Bioanalytical Chemistry**

**Electronic Supplementary Material**

**A novel Ionic Liquid-based Approach for DNA and RNA Extraction simplifies Sample Preparation for Bacterial Diagnostics**

Johanna Kreuter^a*^, Katharina Bica-Schröder^b^, Ádám M. Pálvölgyi^b^, Rudolf Krska^c,d^, Regina Sommer^e*^, Andreas H. Farnleitner^f,g*^, Claudia Kolm^a,f*^, Georg H. Reischer^a*^

^a^ TU Wien, Institute of Chemical, Environmental and Bioscience Engineering, Working Area Molecular Diagnostics 166/5/3, IFA Tulln, Tulln, Austria.

^b^ TU Wien, Institute of Applied Synthetic Chemistry, Research Group for Sustainable Organic Synthesis and Catalysis, Vienna, Austria.

^c^ University of Natural Resources and Life Sciences Vienna (BOKU), Department of Agrobiotechnology (IFA-Tulln), Tulln, Austria.

^d^ Institute for Global Food Security, School of Biological Sciences, Queen’s University Belfast, Northern Ireland, United Kingdom.

^e^ Medical University Vienna, Institute for Hygiene and Applied Immunology, Unit Water Hygiene, Vienna, Austria.

^f^ Karl Landsteiner University of Health Sciences, Division Water Quality and Health, Krems, Austria.

^g^TU Wien, Institute of Chemical, Environmental and Bioscience Engineering, Research Group for Microbiology and Molecular Diagnostics 166/5/3, Vienna, Austria.

^*^ ICC Interuniversity Cooperation Centre Water & Health, Vienna, Austria ([www.waterandhealth.at](http://www.waterandhealth.at)).

+ Corresponding author: [georg.reischer@tuwien.ac.at](mailto:georg.reischer@tuwien.ac.at)

**Contents**

**Table S1** Oligonucleotide sequences for qPCR

**Table S2** Mean lysis efficiencies (in %) for tested bacterial strains, compared to RNA extraction with kit

**Fig. S1** Microscopic pictures of freshly grown bacterial cell suspensions of periopathogens used for extraction

**Table S1** Oligonucleotide sequences for qPCR

| *Assay* | *Oligonucleotide* | *Sequence 5’-3’* | *References* |
| --- | --- | --- | --- |
| 16S qPCR | 8F | AGAGTTTGATCCTGGCTCAG | [1] |
|  | 338R | TGCTGCCTCCCGTAGGAGT | [2] |
| PI qPCR | Forward | TCCACCGATGAATCTTTGGTC | [3] |
|  | Reverse | ATCCAACCTTCCCTCCACTC |  |
|  | Probe | FAM-CGTCAGATGCCATATGTGGACAACATCG-TAMRA |  |

**Table S2** Lysis efficiencies (in %) for tested bacterial strains, compared to RNA extraction with kit as 100%. Data shown are mean values from three biological replicates

| *Sample* | ***Mean Lysis Rate [%]*** | *Min [%]* | *Max [%]* |
| --- | --- | --- | --- |
| *P. intermedia* [C_2_mim][OAc] | **194** | 151 | 257 |
| *P. intermedia* Tris buffer control | **0.2** | 0.1 | 0.2 |
| *A. actinomycetemcomitans* [C_2_mim][OAc] | **404** | 284 | 637 |
| *A. actinomycetemcomitans* Tris buffer control | **90** | 76 | 121 |
| *P. gingivalis* [C_2_mim][OAc] | **702** | 407 | 1066 |
| *P. gingivalis* Tris buffer control | **48** | 34 | 59 |
| *T. denticola* [C_2_mim][OAc] | **773** | 522 | 1195 |
| *T. denticola* Tris buffer control | **3.0** | 1.5 | 6.5 |
| *T. forsythia* [C_2_mim][OAc] | **136** | 111 | 166 |
| *T. forsythia* Tris buffer control | **2.4** | 1.6 | 3.2 |


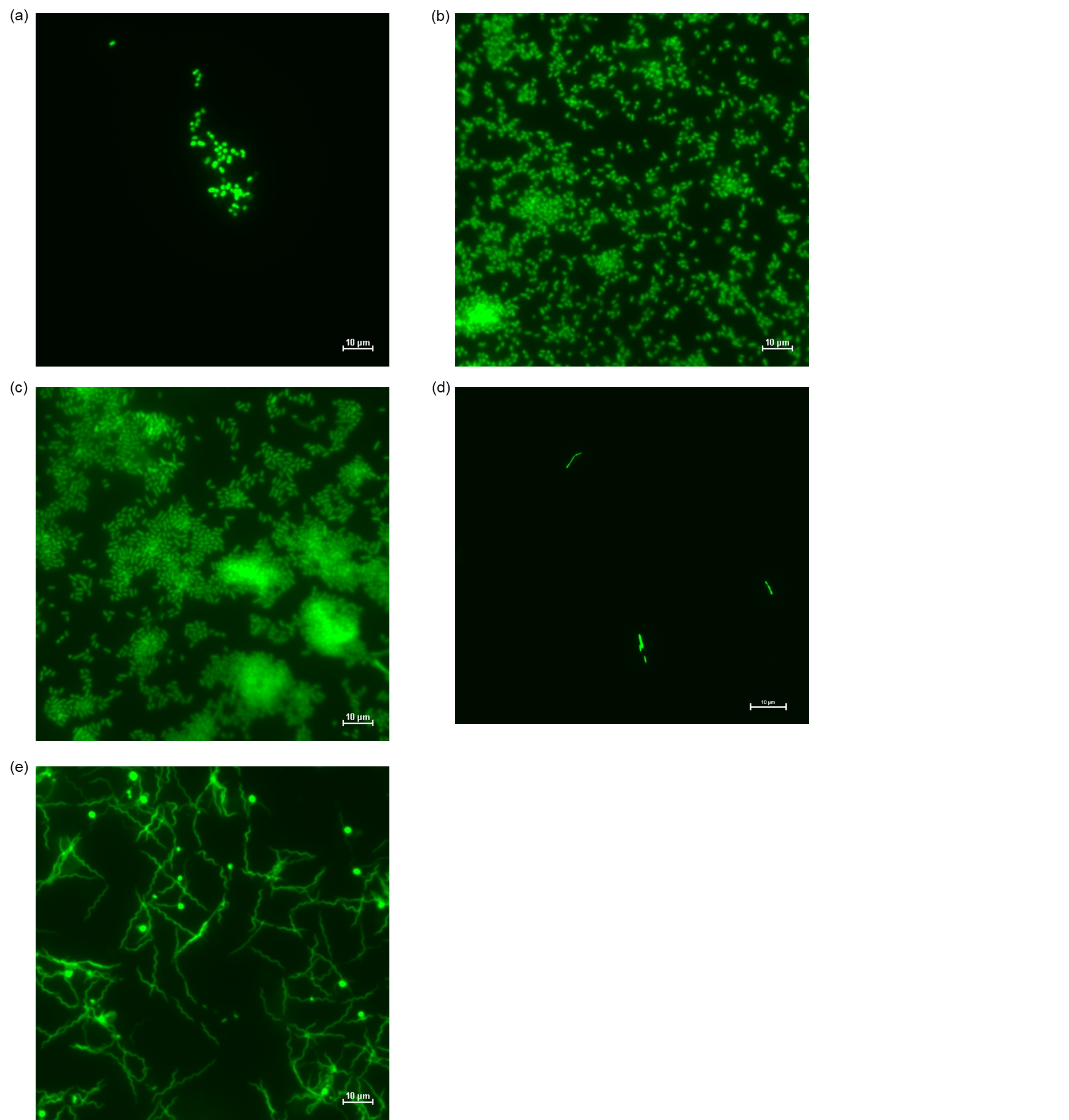


**Fig. S1** Microscopic pictures of freshly grown bacterial cell suspensions of periopathogens used for extraction. Samples were fixed with 4% paraformaldehyde, filtered on 0.22 µm polycarbonate filters, stained with SYBR Gold, and imaged under an epifluorescence microscope (scale bar: 10 µm). (a) P. intermedia; (b) A. actinomycetemcomitans; (c) P. gingivalis (d) T. denticola (e) T. forsythia

**References**

1. Edwards U, Rogall T, Blöcker H, Emde M, Böttger EC. Isolation and direct complete nucleotide determination of entire genes. Characterization of a gene coding for 16S ribosomal RNA. Nucleic Acids Res. 1989;17(19):7843-53; <https://doi.org/10.1093/nar/17.19.7843>.
2. Etchebehere C, Tiedje J. Presence of two different active nirS nitrite reductase genes in a denitrifying Thauera sp. from a high-nitrate-removal-rate reactor. Appl Environ Microbiol. 2005;71(9):5642-5; <https://doi.org/10.1128/aem.71.9.5642-5645.2005>.
3. Kuboniwa M, Amano A, Kimura KR, Sekine S, Kato S, Yamamoto Y, et al. Quantitative detection of periodontal pathogens using real-time polymerase chain reaction with TaqMan probes. Oral Microbiology and Immunology. 2004;19(3):168-76; <https://doi.org/10.1111/j.0902-0055.2004.00135.x>.
